# Variation in Age and Size in Fennoscandian Three-Spined Sticklebacks (*Gasterosteus aculeatus*)

**DOI:** 10.1371/journal.pone.0080866

**Published:** 2013-11-19

**Authors:** Jacquelin DeFaveri, Juha Merilä

**Affiliations:** Ecological Genetics Research Unit, Department of Biosciences, University of Helsinki, Helsinki, Finland; Swansea University, United Kingdom

## Abstract

Average age and maximum life span of breeding adult three-spined sticklebacks (*Gasterosteus aculeatus*) were determined in eight Fennoscandian localities with the aid of skeletochronology. The average age varied from 1.8 to 3.6 years, and maximum life span from three to six years depending on the locality. On average, fish from marine populations were significantly older than those from freshwater populations, but variation within habitat types was large. We also found significant differences in mean body size among different habitat types and populations, but only the population differences remained significant after accounting for variation due to age effects. These results show that generation length and longevity in three-spined sticklebacks can vary significantly from one locality to another, and that population differences in mean body size cannot be explained as a simple consequence of differences in population age structure. We also describe a nanistic population from northern Finland exhibiting long life span and small body size.

## Introduction

Age at first reproduction as well as life span are life history variables that are not only important for individual fitness through their effects on lifetime reproductive output (e.g. [1]), but also for population dynamics and demographic structure – and thereby also evolution – of wild populations [2]. Apart from being parameters of central importance in studies of evolution of individual life histories and population dynamics, life span and its intrinsic, as well as extrinsic, determinants continue to attract interest in the context of research focused on aging and senescence (e.g. [3], [4]). Hence, studies focused on population age structure can be interesting for many different reasons.

Knowledge of the population age structure is also critically important for empirical studies of effective population size (*N_e_*). For instance, the population genetic approaches developed to estimate *N_e_* from temporal changes in allele frequencies in neutral loci can be sensitive to biases caused by overlapping generations (e.g. [5]). Hence, some knowledge of generation length is needed. The importance of this was nicely illustrated by Cuveliers et al. [6] showing that the *N_e_* estimates for sole (*Solea solea*) changed over time as a response to reduced generation length caused by fisheries-induced shifts towards earlier maturation with time. Hence, since the generation time can be approximated from the average age of breeding adults in the population [7], knowledge about spatial and temporal variation in population age distribution can aid studies of *N_e_*.

Numerous studies have provided estimates of ages at first reproduction, maximum age and life span of three-spined sticklebacks (*Gasterosteus aculeatus*) in various geographical locations and different habitats (reviewed in [8]; see also: [9]; [10]; [11] for more recent case studies and [12], [13], [14] for earlier reviews). Comparison of maximum life span among anadromous, lacustrine and stream-dwelling populations revealed that 45% of the stream-dwelling populations were annual, whereas anadromous populations were rarely so [8]. However, as pointed out by reviews of three-spined stickleback life-histories [8], [15], the data on age and maximum life span is of very heterogeneous quality. For instance, most estimates of population age structure are based on size-frequency plots rather than histologically determined age. Although it has been suggested that age-class modes are clearly structured in sticklebacks [15], it is known that various ecological factors such as productivity, predation and parasitism can also have interactive effects on body size [11], [15]. Furthermore, although it appears that low-latitude populations tend to be almost invariably short-lived as compared to high-latitude populations, which can be either short- or long-lived [8], information about northern European populations is too scarce to confirm this trend. In fact, most (96%) of the available (n = 26) estimates are from latitudes below 60 degrees north [8]. As such, it is as yet unclear whether high-latitude populations in Fennoscandia breed primarily at age two years as they do in Alaska ([15] p. 592).

The aim of this study was to investigate age and size structure of adult three-spined stickleback populations in different parts of Fennoscandia. In particular, we were interested in determining if there are any marked differences in mean age (i.e. generation length; [7]), and thus life span, of sticklebacks among these localities. To this end, we collected sticklebacks from eight different localities, representing three different habitat types (*viz*. marine, lake and pond locations), and determined their age with skeletochronological methods. Apart from advancing our basic understanding of variation in important life history traits in this species, the results should be useful for studies seeking to estimate effective size of stickleback populations in different habitats using genetic methods (cf. [5]).

## Methods

### Ethics statement

This study was carried out in strict accordance with the Finnish and Swedish legislation and the fish were collected under appropriate national fishing licenses of the respective countries. The research described in this paper does not involve animal experiments according to The Act of Animal Experimentation (FINLEX 497/2013; http://www.finlex.fi/fi/laki/alkup/2013/20130497). The fish were sacrificed by an overdose of MS-222 (tricaine methanesulfonate) immediately upon their capture. Hence, suffering before anesthesia was minimal.

### Sampling

**Figure 1:**
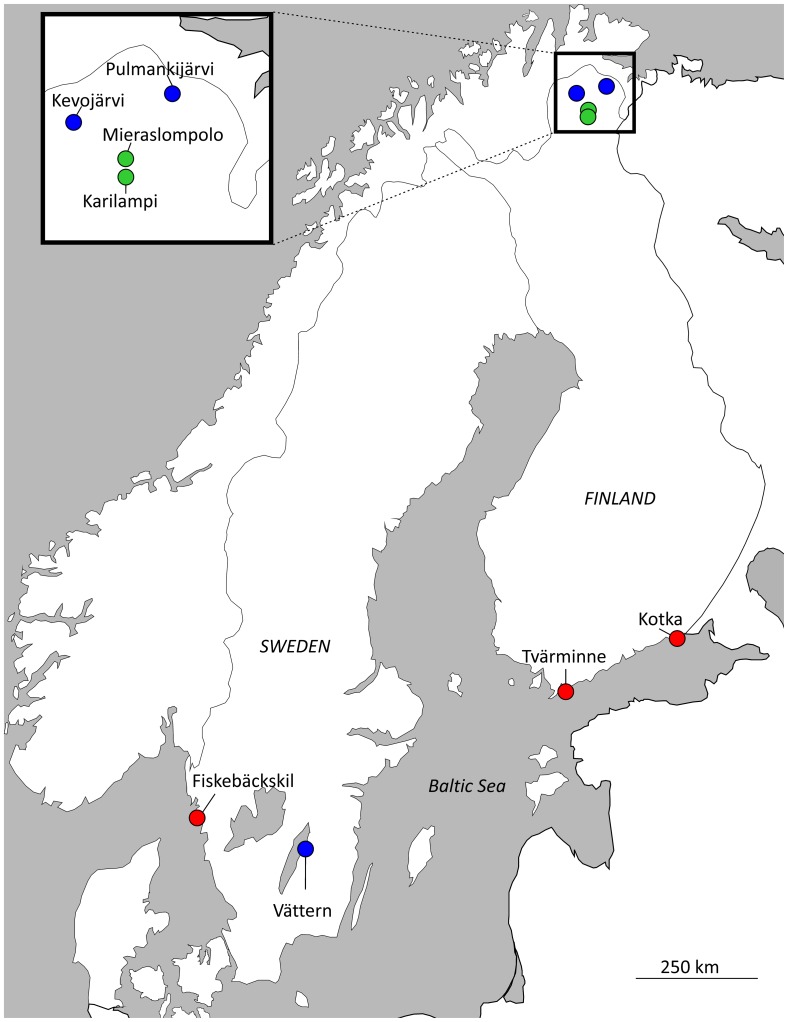
Location of the eight Fennoscandian sampling sites used in this study. The insert shows the location of the four northern Finnish sites in more detail. Red  =  marine, blue  =  lake, green  =  pond.

The samples for this study were collected from eight different Fennoscandian sites shown in Fig. 1. Three of the sites were marine locations in the Baltic and North Seas, and three were large lakes (Table 1). All of these marine and lake populations harbor diverse fish fauna, including many stickleback predators such as European perch (*Perca fluviatilis*), brown trout (*Salmo trutta*), pike (*Esox lucius*) and Atlantic salmon (*Salmo salar*). The remaining two sampling locations were isolated ponds lacking predatory fish, with the possible exception of brown trout in Karilampi (Table 1).

**Table 1:** Descriptive information about the study sites and samples.

Location	Coordinates	Habitat	Collection date	Females (n)	Males (n)	Total (n)
Fiskebäckskil	58°24**′**N, 11°47**′**E	Marine	June 2003	18	20	28
Kotka	60°27**′**N, 26°55**′**E	Marine	5 June 2003	15	15	30
Tvärminne	59°50**′**N, 23°12**′**E	Marine	10 June 2003	14	16	30
Kevojärvi	69°45**′**N, 27°00**′**E	Lake	26 June 2003	15	15	30
Pulmankijärvi	69°58**′**N, 27°58**′**E	Lake	30 June 2003	8	22	30
Vättern	58°54**′**N, 14°24**′**E	Lake	23 July 2003	24	2	26
			10 June 2004	5	0	5
Mieraslompolo	69°34**′**N, 27°14**′**E	Pond	3 July 2003	16	14	30
Karilampi	69°33**′**N, 27°14**′**E	Pond	26 June 2003	22	8	30
Total (n)				137	102	239

n =  sample size.

The fish were caught with seine nets in 2003; the sample from Lake Vättern was supplemented with fish caught in 2004 (Table 1). We sought to age approximately similar numbers reproductive females (n = 15) and males (n = 15) from each of the localities. However, due to long-term preservation (age determinations were carried out in 2005 and 2011) in formalin, some of the samples were too degraded to accurately determine the age. In total, age was successfully determined for 239 fish (Table 1).

Aged individuals represented a random sample of mature individuals from a given population. Sex and maturity of all fish was verified with gonadal inspection. In the case of fish collected from lake Vättern in 2003, most fish showed clear signs of growth after the appearance of the last annuli, indicating a of period arrested growth (winter). Hence, these fish can be expected to be larger than indicated by their chronological age from growth annuli, and this needs to be taken into account when interpreting size data for the Vättern population. Standard length (from tip of the nose to the tail base; see Fig. 1 in [16]) was taken with calipers/photographs and used as a proxy of body size. Using 77 fish for which the centroid size – a multivariate morphometric measure of size [17], [18] – was available, we confirmed that the correlation with these two measures was nearly perfect (*r* = 0.99, P<0.001).

### Age determination

Age determinations were carried out using standard skeletochronical analyses, which are based on the realization that seasonal periods of arrested growth leave clearly defined annuli (‘lines of arrested growth’) on bony structures [19], [20]. In short, ages were determined by counting annuli either in sagittal otoliths, pelvic (or dorsal) spines or fin rays following [19] and [12]. The otoliths were cleaned mechanically with forceps and examined while submerged in water on a dark background, untreated, using transmitted light microscopy to visualize the opaque and transparent annuli [21]. With many of the samples, the aging from otoliths was found to be difficult or impossible due to sample degradation. However, for a subsample of fish, we verified that reading from otoliths, spines and fin rays gave similar age estimates. The use of different tissues for age determination was also used to distinguish genuine annuli from false annuli. False annuli can be produced during repeated reproductive cycles in the same summer, but these are usually much weaker and irregular as compared to true annuli produced during winter.

Fins and spines were first cut as near to the base of the fin/spine as possible, cleaned carefully from extra tissue, treated with 1,2-Propanediol to gain better contrast and then air-dried. Fins/spines were then stained with a neutral red solution (with acetic acid) and the annuli were evaluated under microscope with 30–100× magnification. From this data we estimated age of the individual fish as the number of annuli. Fish with one annuli would have been born the year before, and therefore be in the second calendar year (yearling), whereas fish with two annuli would be in the third calendar year (a two year old fish), and so on. Under the assumption of random sampling and that our snapshot samples (sampling conducted only in one year) are representative, we estimated the (conservative) maximum age of fish ( = life span) at each site as the age of the oldest individual in the sample. This is of course only a minimum estimate given the relatively small sample sizes. Nevertheless, the figures should at least give tentative indications about the age structure and life span in different sites.

### Statistical analyses

Generalized linear models were used to analyze the age and size data. Individual age was fitted as a Poisson distributed response variable, with habitat type, sex and the population (nested within habitat type) as factors. Interactions of habitat*sex and location*sex were also fitted, but because they were non-significant (P≥0.49) they were dropped and not reported. The results were qualitatively similar if age was fitted as a normally distributed response variable.

In order to investigate how age influences mean body size, we first fitted a model where individual size (a normally distributed response variable) was modeled as a function of habitat type (fixed factor), sex (fixed factor) and population (random factor nested within habitat type). We then repeated the analysis by including age as a covariate to see whether the size differences persisted after accounting for variation due to age. If the habitat or population differences in size are solely due to age differences among habitats and populations, there should be no significant habitat or population effects after accounting for age effects. In these models, we also included sex*habitat and sex*population (within habitat type) interactions but dropped them from the final model if non-significant.

All statistical analyses were conducted with JMP 10 Pro (ver. 10.0.2d1) statistical package (SAS Institute Inc.) run on Apple Macintosh platform.

### Data accessibility

All the data behind this publication has been submitted to a Dryad archive: [DOI: doi:10.5061/dryad.d2vh0].

## Results

**Figure 2:**
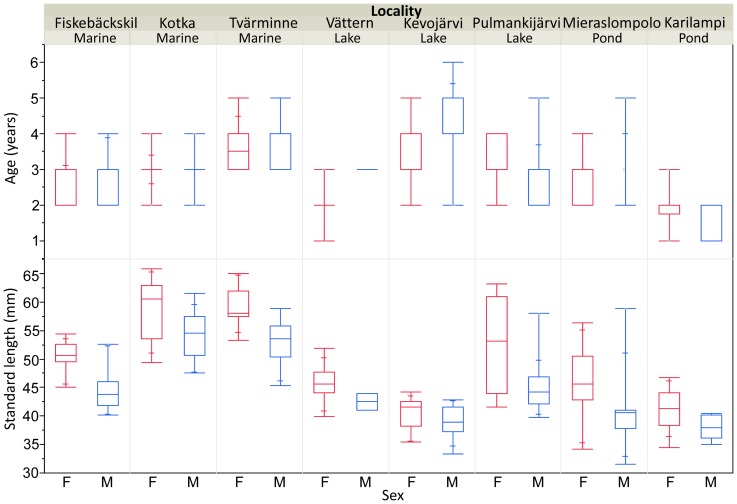
Box-plots of (a) age and (b) standard length of female and male three-spined sticklebacks in eight different localities arranged by habitat type. The boxes depict the first and third quartiles and band within boxes the median. Maximum and minimum data values are depicted by whiskers with wide bars, the narrower bars depicting 10% and 90% quartile ranges. For sample sizes, see Table 1.

The distribution of ages for the two sexes in each locality is plotted in Fig. 2a. A generalized linear model fitted to the data revealed that there were significant differences in mean age among habitat types (LR ChiSquare  = 11.98, df = 2, P = 0.0025), chiefly due to the contrast between marine (higher mean age) and pond (lower mean age) populations (Fig. 2a). However, as revealed by the same analysis and inspection of values in Fig. 2a, population differences within habitat types were also large (LR ChiSquare  = 20.34, df = 5, P = 0.0011). The mean age did not differ between sexes (LR ChiSquare  = 0.0003, df = 1, P = 0.98; Fig. 2a).

Notably, one-year old individuals (n = 9) were encountered only in two of the freshwater populations, whereas the minimum age in all other populations was two to three years (Fig. 2a). Maximum ages ranged from three (n = 2) to six years (n = 1), with no apparent pattern across the habitat types (Fig. 2a).

**Figure 3:**
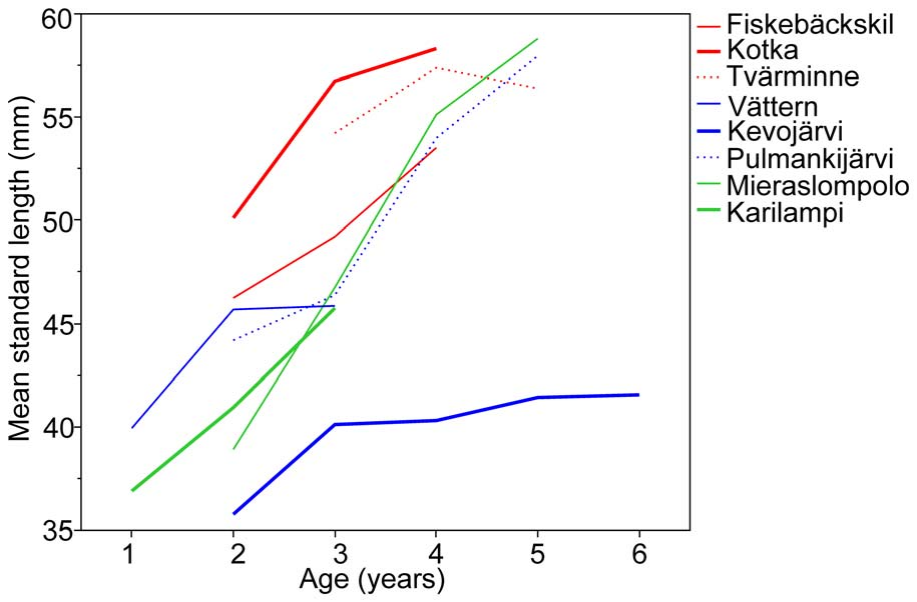
Mean standard length in different localities as a function of age.

The average size of individuals in a given population was highly variable (Fig. 2b). Mean size differed significantly between habitat types (F_2,4.98_ = 6.15, P = 0.045), mostly because the fish from marine localities were larger than those from freshwater localities (Fig. 2b). Likewise, females were on average larger than males (F_1,232.3_ = 60.97, P<0.001; Fig. 2) in all localities (variance component [±S.E.] due to sex*locality interaction: 1.22±1.66; 3.4% of variance accounted for) and in all habitats (sex*habitat: F_2,230.3_ = 0.64, P = 0.53). However, the within-habitat type variation in mean size was substantial as seen in Fig. 2b, and in the fact that the variance component due this effect accounted for 48.8% of total variance. Adding individual age as a covariate into this model revealed a significant positive effect of age on size (F_1,232.6_ = 117.20, P<0.001), and rendered the habitat effect non-significant (F_2,5.01_ = 3.59, P = 0.11). Hence, the size differences among habitats were at least partly caused by habitat differences in age. However, even after accounting for age effects, the among-locality variance component was still large (62.6% variance explained), meaning that age differences cannot explain all the variation in body size. This is perhaps best illustrated by plotting the age-specific mean sizes across different populations, which reveals that for all given ages, the fish from Lake Kevojärvi are smaller than those from all other populations (Fig. 3). This stunted growth of the Kevojärvi fish is also apparent from Figs. 2a and b: while having similar mean size to fish from Karilampi pond (smallest mean size in Fig. 3), the Kevojärvi fish have the largest mean age among the population examined here (Fig. 2a).

## Discussion

We found considerable variation in mean age of breeding sticklebacks across different sampling sites. Although there were some indications that this variability was partly associated with habitat, the variance among sites within habitats was large. Not surprisingly, there was little indication of sex differences in mean age and/or longevity across the sites or habitats. Likewise, while the size of individuals within populations increased with age, there were some marked age-independent differences in mean size of individuals among populations. For instance, while the fish from the pond Karilampi did not differ in mean size from fish in the nearby Lake Kevojärvi, there was roughly a two-fold difference in mean age of individuals between these localities. Hence, marked size-at-age differences among populations were obvious. In what follows, we discuss these findings in light of what was previously known about age structure and life span of three-spined sticklebacks, as well as the potential implications of our findings to future studies of Fennoscandian three-spined sticklebacks.

The age at which sexual maturity in three-spined sticklebacks is reached varies from one (e.g. [21]–[25]) to several years (e.g. [8], [15], [26]). In our data, we encountered very few one year old reproductive individuals. This suggests that sexual maturity in Fennoscandian locations seldom occurs before sticklebacks are on their third calendar year (i.e. ‘2 years old’). This matches Aneer's [26] observation, according to which Baltic Sea sticklebacks do not mature before 15 months old, translating to the conclusion that fish born in a given year (June-July) are not ready to breed in the year after, as the breeding season comes to an end in July [26]. This is of course not to say that maturation could not occur also at an earlier age if conditions for development and growth are extremely favorable. For instance, in the laboratory Baltic Sea three-spined sticklebacks can mature and breed before they have reached an age of one year, even much earlier (J. DeFaveri, personal obs.; see also [27]). However, in light of the age data collected from a wide range of localities in Fennoscandia, it appears that most wild fish in these high-latitude populations mature earliest at an age of two years. In fact, supplementing the data from Appendix 6.1 of [8] with data from the current study, the probability of maturing at an age of two years (in contrast to maturation at an age of one year) is a positive function of latitude across populations in Europe (Generalized Linear Mixed Model: b = 0.51±0.21, n = 34, LR Chi-Square  = 18.28, P<0.001), even after controlling for the significant (LR Chi-Square  = 13.75, P = 0.001) effect of habitat type. Hence, across its European range, age at maturity of sticklebacks appears to be delayed both by anadromous life-style and increasing latitude.

As to the maximum age and life span, we observed five to six year old individuals in four of the eight locations. In Baker's compilation of literature data [8], there were only two European locations where maximum recorded ages were as high as four and five years, respectively. In this view, our new data from the high-latitude populations complements the picture and suggests that three-spined sticklebacks from northern Europe often reach ages well beyond four years.

Over its entire distribution range, there is considerable variation in three-spined stickleback lifespan. While there are populations that are effectively annual (i.e. breed at an age of one year and die thereafter) such as those living in England [28], [29] and France [25], there are also populations in British Colombia where individuals frequently reach ages of up to six years [10], and sometimes even eight years [12]. Although most three-spined sticklebacks populations – including the ones studied here – reside in between these extremes [8], one should note that with our sample sizes of ca. 30 individuals per population, it is quite likely that some even older individuals were missed. For instance, screening through 100 of the largest individuals in their Drizzle Lake samples, Gambling & Reimchen [10] were unable to find any individuals older than seven years, although these had been earlier recorded from this lake from a sample of 492 individuals. Hence, the maximum age estimates in our data are likely to be conservative, and additional sampling would most likely increase these estimates by a year or two.

Across its distribution range, the average size of breeding threespine sticklebacks varies from about 31 mm to 90 mm in standard length, and the mean size is usually larger in anadromous as compared to freshwater populations [8]. However, within-habitat type variation is also large. This is nicely illustrated by the occurrence of both giant-sized (sometimes >100 mm in SL; [10], [12]) and dwarf-sized (maturing at sizes as small as 23 mm in SL; [11]) in freshwater habitats. The results of this study agree with this general pattern: fish collected from marine locations were generally larger than those from freshwater locations, but much of these habitat-specific differences disappeared when differences in age were controlled for. Yet, variation among localities within habitat types persisted even after controlling for age variation. Such population specific differences in size-at-age may be traced ultimately to either genetic or environmental differences (or both) in patterns of growth. Disentangling these alternatives requires common garden breeding experiments, which would provide a way to further explore, for example, the interesting case of the stunted growth in Kevojärvi fish pictured in Fig. 3. Hence, although generalizations about the patterns of geographic and habitat specific variation in body size attained by sticklebacks are hard to make given the wide variety of life-histories, re-analysis of Baker's data [8] – supplemented with data from this study – shows that the minimum size at maturation in three-spined sticklebacks increases with increasing latitude, but this effect is not significant (b = 0.28±0.17, F_1,32_ = 2.73 P = 0.10) when controlling for the significant effect of habitat type (F_3,32_ = 8.78, P<0.001). Yet, it is worth re-emphasizing the fact that deviations from these broad scale patterns – including the effect of habitat type – are frequent as illustrated also by the wide range of variation discovered in this study. Furthermore, the results of the present study in respect to habitat type effects are at best tentative due to relatively small sample sizes (only two pond populations): more populations from each of the different habitat types are required generalize our findings.

The flat size-at-age distribution of the Kevojärvi fish discovered in this study resembles closely that of the giant three-spined sticklebacks in British Colombia [10], [12]. As pointed out by Reimchen [12], the extended longevity and the small yearly increments in size render the use of size-frequency distributions for aging unreliable in populations where old ages are frequently reached. Unfortunately, age determinations based on otolith or spine annuli are time consuming, and only few stickleback studies (e.g. [10], [12], [21]) have applied this methodology. To this end, our study (n = 8 populations, 239 individuals) and that of Gambling & Reimchen ([10]; n = 13 populations, 65 individuals) are perhaps spatially and numerically the most comprehensive so far, and illustrate how histological age determinations can help us to refine our understanding of fish life histories. Further insights into such studies can be gained by inclusion of isotope ratio analyses which can inform us about individual variation in habitat utilization, growth and life history strategies associated with variation in age [30].

The results of this study are not only relevant from the life history evolution perspective, but also from the perspectives of conservation biology and genetics. The evolutionary potential and ability of populations to persist in the face of environmental changes can be critically dependent on the amount of genetic variation they harbor [31]. The amount of genetic variability a population can sustain depends in turn very much on its effective size (*N_e_*): the smaller the *N_e_*, the faster the loss of variability due to genetic drift [31]. Hence, estimating and understanding *N_e_* of wild populations has become an increasingly important activity in the realm of conservation genetics [32]. Various methods and approaches have been developed to estimate *N_e_* from molecular data, several of which estimate *N_e_* based on allele frequency fluctuations among generations. However, these methods can be sensitive if the underlying assumption of non-overlapping generations is violated (e.g. [5], [33]). Hence, for species and populations with overlapping generations, the knowledge about population age structure is critical as it allows for either correcting (e.g. [34]) or alleviating this problem by designing appropriate generation length-dependent sampling schemes. To this end, the site-specific average ages should give a fairly good estimate about the variation in average lifespan among Fennoscandian populations, and hence, also about their generation intervals [7]. Given the large variation in life histories of three-spined sticklebacks, it not unthinkable that there are also large differences in their effective population sizes. In this perspective, the information put forth in this study should aid in their estimations by using appropriate corrections and sampling schemes.

In conclusion, the results from this study suggest that there is considerable variation in average age and size of breeding three-spined sticklebacks in Fennoscandia. While habitat effects on both traits are clear, they are relatively weak and, as in the case of body size, largely caused by habitat differences in age. Although further studies utilizing larger sample sizes and repeated sampling of the same sites could help to get a more refined picture of variation in population age structure, it can be concluded that three-spined stickleback generation lengths in Fennoscandia range from approximately two to four years. Hence, studies aiming to obtain reliable estimates of effective population sizes based on temporal variation in allele frequencies under ‘3 to 5 generation separation criteria’ (e.g. [5], p. 794) would need to collect samples at least over of period of six to 12 years.
